# Transcriptome Analysis Reveals Genes Responsive to Three Low-Temperature Treatments in *Arabidopsis thaliana*

**DOI:** 10.3390/plants13223127

**Published:** 2024-11-06

**Authors:** Bricia Ruiz-Aguilar, Natalia B. Torres-Serrallonga, María Azucena Ortega-Amaro, Arianna Duque-Ortiz, Cesaré Ovando-Vázquez, Juan Francisco Jiménez-Bremont

**Affiliations:** 1 Laboratorio de Biotecnología Molecular de Plantas, División de Biología Molecular, Instituto Potosino de Investigación Científica y Tecnológica, A. C., San Luis Potosí, S.L.P. 78216, Mexiconataliatose@gmail.com (N.B.T.-S.);; 2 Coordinación Académica Región Altiplano Oeste, Universidad Autónoma de San Luis Potosí, Salinas de Hidalgo 78600, Mexico; 3 Laboratorio de Bioinformática e Inteligencia Artificial, CONAHCyT–Centro Nacional de Supercómputo, Instituto Potosino de Investigación Científica y Tecnológica, A.C., San Luis Potosí, S.L.P. 78216, Mexico

**Keywords:** *Arabidopsis thaliana (L)*, cold stress, differential gene expression, RNA-seq, RNA secondary structure unwinding

## Abstract

Cold stress impedes the growth and development of plants, restricts the geographical distribution of plant species, and impacts crop productivity. In this study, we analyzed the *Arabidopsis thaliana* transcriptome to identify differentially expressed genes (DEGs) in 14-day-old plantlets exposed to temperatures of 0 °C, 4 °C, and 10 °C for 24 h, compared to the 22 °C control group. Among the top 50 cold-induced genes at each temperature, we identified 31 genes that were common across all three low temperatures, with nine genes common to 0–4 °C, eight genes to 4–10 °C, and two genes to 0–10 °C. Using q-RTPCR, we analyzed selected genes at 24, 48, and 72 h under the three low temperatures. Our data revealed that genes, such as galactinol synthase 3 (*Gols3*, At1g09350), *CIR1* (At5g37260), *DnaJ* (At1g71000), and At5g05220 (unknown function), exhibited the highest expressions at 0 °C and 4 °C throughout all time points. We also studied genes from the UDP-glycosyltransferase (UGT78) family, including At5g17030 (*D3*), At5g17040 (*D4*), At5g17050 (*D2*), and At1g30530 (*D1*), which showed increased expression at low temperatures compared to plantlets at 22 °C for 24 h. Gene ontology analysis revealed that DEGs highly enriched were found in biological processes such as “RNA secondary structure unwinding” and “rRNA processing” induced at the three low temperatures, whereas processes related to photosynthesis were repressed. Our findings indicated upregulation in the expression of four RNA helicases (*RH13, RH48, RH32*, and *RH29*), belonging to the “RNA secondary structure unwinding” category, mainly at 0 °C and 4 °C. This study provides valuable information on the molecular mechanisms that activate *Arabidopsis thaliana* in its early response to these three low temperatures.

## 1. Introduction

Extreme temperatures are considered to be among the most severe abiotic stressors, causing significant limitations on the growth and development of plants. As sessile organisms, plants have evolved mechanisms to tolerate a wide range of abiotic stresses, and one of the most significant challenges they face is coping with cold stress. Cold stress can be categorized into chilling and freezing stresses. Chilling stress typically occurs within the temperature range of 0 °C to 15 °C, while freezing stress occurs at temperatures below 0 °C [1].

Studies have indicated that plants can suffer various detrimental consequences due to low temperatures, including tissue damage, wilting, reduced growth rate, chlorosis, and accelerated senescence, among other adverse effects [2]. In plant cells, low temperatures impact membrane rigidification, protein destabilization, and reduced enzyme activities. This results in photoinhibition, compromised photosynthesis, reduced cell division, and substantial damage to cell membranes [3,4]. Moreover, cold stress induces the formation of secondary structures in RNA, directly affecting gene expression and protein synthesis in plant cells [3,5]. To withstand cold stress, plants adapt by reprogramming their gene expression and altering their metabolism [1]. This adaptation involves the production of osmolytes such as soluble sugars, sugar alcohols, low-molecular-weight nitrogenous compounds, and the accumulation of diverse proteins [6]. Among the accumulated proteins are cold-regulated proteins (CORs), chaperones, dehydrins, heat shock proteins (HSP), antifreeze proteins, pathogen-related (PR) proteins, detoxification enzymes, mRNA-binding proteins, transporters, lipid-transfer proteins, proteinase inhibitors, and enzymes involved in the biosynthesis of osmoprotectants [1,7,8,9].

Upon sensing cold temperatures, plants initiate their molecular responses, inducing many genes that trigger stress responses to protect their cells. At the same time, another group of genes, such as those involved in photosynthesis, is downregulated as plants adapt to these growth conditions [10]. In response to cold stress, cells express response genes through transcription factors such as ICE1 and CBF. The *ICE1* (Inducer of CBF Expression 1) gene encodes a MYC transcription factor activated by MAPK signaling cascades [11]. It induces CBF (C-repeat binding factor) genes from the AP2/ERF family, which bind to CRT/DRE (C-Repeat/Dehydration Response Element) cis-elements in the promoters of COR genes. In turn, COR genes encode proteins with cryoprotective effects, such as COR15A and COR15B, which help protect and stabilize chloroplasts during freezing [12].

At lower temperatures, RNA structures tend to become more stable, potentially leading to the formation of secondary structures that could hinder translation and/or affect the functions of these RNAs. RNA helicases, functioning as molecular motors, play a pivotal role in rearranging RNA secondary structures and are implicated in a diverse array of cellular processes associated with RNA metabolism. These processes encompass RNA splicing, transcription, translation, RNA degradation, and ribosome biogenesis [13,14]. Therefore, in periods of cold stress, helicases could play a role in regulating RNA metabolism within plant cells, potentially enhancing translation at lower temperatures.

Tolerance to cold stress varies among plant species. For instance, tropical and subtropical plants tend to be more sensitive to low temperatures. In contrast, specific species like *Arabidopsis thaliana* (*Arabidopsis*), *Spinacia oleracea* (spinach), *Avena sativa* (oat), *Hordeum vulgare* (barley), *Triticum aestivum* (wheat), and *Secale cereale* (rye), among others, have developed greater tolerance to cold stress through specialized mechanisms, enabling them to grow and thrive in cold conditions [1]. The plant *Arabidopsis thaliana* has several traits that make it an ideal model for studying molecular responses to challenging conditions like drought, salinity, and extreme temperatures. These traits include being a small, flowering plant with a short life cycle that is easy to handle in the laboratory, as well as having a sequenced genome that shares many stress response mechanisms with other plant species. Notably, *Arabidopsis thaliana*, a plant native to Eurasia and Africa, is naturally adapted to grow in cold temperatures in those regions. This model plant has been used to understand the molecular and physiological basis of cold tolerance [15,16,17,18].

Among cold-tolerant plant species, these have developed physiological and molecular adaptations to enhance their tolerance to low temperatures, including the regulation of gene expression. In this study, with the aim of examining the differential gene expression that occurs in the plant when exposed to cold stress, we conducted RNA-seq to analyze the transcriptional profiles in *A. thaliana* plantlets, when they were subjected to three low temperatures (0 °C, 4 °C, and 10 °C) for 24 h. Bioinformatics analyses were performed to identify enriched Gene Ontology terms shared under the examined cold stress conditions. We then selected several genes that showed upregulation in the RNA sequencing data, which were subsequently analyzed by qRT-PCR to assess their expression at the three low temperatures.

## 2. Material and Methods

### 2.1. Plant Material and Experimental Conditions

*Arabidopsis thaliana* is a small annual weed commonly used as a model organism due to its compact genome, short life cycle, ease of cultivation, and high seed yield. Its life cycle can range from as short as 6–8 weeks to as long as 10 weeks. The distribution of this plant is limited by very low temperatures in spring and autumn, as well as by high temperatures (>22 °C) and low precipitation in summer [19]. The leaves of *A. thaliana* are arranged in a compact rosette, with inflorescences that produce a large number of siliques [20]. In this study, the seeds of *Arabidopsis thaliana* ecotype Columbia 0 (Col-0) were surface-sterilized by immersing them in a solution of 20% (*v*/*v*) household bleach (approximately 2.7% sodium hypochlorite) for 5 min, after which they were rinsed five times with sterile distilled water. The sterilized and stratified seeds were vernalized for two days at 4 °C. Afterward, they were germinated and cultivated in vitro on Petri plates containing 0.2× Murashige and Skoog medium (MS) (PhytoTechnology Laboratories^®^, Lenexa, KS, USA), adjusted pH of 7.0, with 0.5% (*w*/*v*) sucrose, and 1% (*w*/*v*) agar. This cultivation was carried out in a chamber with controlled temperature conditions (22 °C ± 1 °C), and a light cycle of 16 h of light (13,000 lux) followed by 8 h of darkness. After growing for two weeks, 14-day-old plantlets were subjected to low temperatures of 0 °C, 4 °C, and 10 °C for 24 h, 48 h, and 72 h, respectively. Control plants were maintained in a growth chamber at 22 °C. After the cold treatments, the plants were harvested, rapidly frozen in liquid nitrogen, and stored at −80 °C for further analysis. Each temperature condition had three biological replicates (n = 3), with each replicate comprising groups of 10 plantlets per Petri plate.

### 2.2. RNA Extraction and Next-Generation Sequencing

Total RNA extraction from plant material subjected to the low-temperature treatments (0 °C, 4 °C, and 10 °C) and as control (22 °C) for 24 h was conducted following the ConcertTM Plant RNA Reagent protocol (Invitrogen Life Technologies, Waltham, MA, USA). The quality of the RNA samples was evaluated using a 1% agarose gel and 0.5x TBE buffer. RNA concentration was determined using a NanoDrop ND-1000 UV–vis spectrophotometer (NanoDrop Technologies Inc., Wilmington, DE, USA).

Each set of samples was analyzed in triplicate. Subsequently, the RNA samples were prepared for shipment to the GENEWIZ company (Burlington, MA, USA) for paired-end RNA sequencing, following the RNAstable^®^ protocol for RNA preservation at room temperature. Sequencing was performed with HiSeq 2500 technology (Illumina, San Diego, CA, USA) in rapid mode with v2 reagents. The readings produced were 250 bp reads per end, according to the manufacturer’s instructions.

### 2.3. Mapping Sequence Reads to the Reference Genome and Gene Hit Counts Extraction

The RNA-seq fragments were mapped to TAIR10 reference genome using Hisat2 v2.2.0 [21], with default parameters and trimming 50 nt on the 3′ end. After trimming, fragments show a median Phred quality score of at least 30. The mapping process results in a mapped ratio of ~95% on average for all the libraries (Supplementary Table S1).

### 2.4. Functional Annotation

Trinotate [22] was used to annotate the functions of all the transcripts per gene in the reference genome. Trinotate helped us to gather the results of Blastn v2.6.0, Blastp v2.6.0 [23], Hmmer v3.1b2 [24], SignalP v4.1 [25], and tmHmm v2.0 [26].

### 2.5. Transcriptome Mapping, and Matrix Counts Generation

Kallisto v0.42.4 [27] was used to map and quantify the RNA-seq fragments, for each library. We obtained an average of ~95% mapping for all the libraries. The quantifications for each library were stored at different tables. Via a custom R script v4.2.0 [28], we gather the Kallisto pseudo-alignments and quantifications into a counts data frame.

### 2.6. Data Availability

Original RNA-seq data is openly available in Gene Expression Omnibus under accession GSE27881.

### 2.7. Analysis of Differentially Expressed Genes in Response to Cold Treatments

Using the counts data frame, we used the edgeR R package v3.42 [29] to perform differential expression analysis. Each low-temperature condition (0 °C, 4 °C, and 10 °C) was compared to the control condition maintained at 22 °C. Transcripts that showed an adjusted *p*-value (false discovery rate, FDR) lower than 0.01 were considered significant in the analysis. Gene Ontology (GO) term enrichment was performed with custom scripts. The enrichment was calculated with a logistic regression model, defining the classes y = 1 − transcripts in the GO term and y = 0 − transcripts not in the GO term. The x input variable, derived from the differential expression analysis, used to calculate the logistic model was defined as x = sign(logFC) · −log10(FDR). The logistic regression model was fitted using the glm function with the binomial density distribution family and the logit link. The two parameters of the resulting logistic model were used to calculate an enrichment score as = sign(enrichment) · −log10(*p*-value_enrichment).

### 2.8. Expression Analysis of the Genes Identified in the RNA-Seq Data Through qRT-PCR

To perform real-time quantitative RT-PCR, we used the StepOne™ Real-Time PCR System (Applied Biosystems (Waltham, MA, USA), catalog number 4376374). Total RNA was extracted from 14-day-old plantlets exposed to temperatures of 0 °C, 4 °C, 10 °C, and 22 °C. To remove any remaining genomic DNA, DNA digestion was performed using the DNAase Turbo enzyme (Ambion, Austin, TX, USA). Subsequently, cDNA synthesis was performed using 1μg of total RNA and the SuperScript™ II Reverse Transcriptase kit (Invitrogen, Carlsbad, CA, USA). The detection of real-time RT-PCR products was conducted following the SYBR^®^ Green Supermix protocol from Applied Biosystems. To conduct the PCR reactions, a 100-ng dilution of the cDNAs was utilized as a template. The PCR cycling conditions consisted of 40 cycles, with each cycle comprising 10 s of denaturation at 95 °C and 30 s of annealing/extension at 60 °C. Following that, melting curves were generated by gradually increasing the temperature from 60 °C to 95 °C in 0.3 °C intervals. The list of genes and their corresponding primers for qRT-PCR assays is available in Supplementary Table S2. The cycle number threshold (Ct value) was employed to calculate relative mRNA expression levels. The Ct value for each target gene was normalized by subtracting the Ct value of the *A. thaliana ubiquitin-5* (*AtUBQ5*, *At3g62250*) gene. The mRNA expression levels were obtained using the 2^−ΔCt^ method [30] compared to the control samples (plants maintained at 22 °C for each period). Each sample was analyzed with three biological replicates, each having their respective technical replicates.

### 2.9. Amplification of the CIR1-I3R Splicing Isoform by RT-PCR

For semiquantitative RT-PCR experiments, 14-day-old *Arabidopsis* plantlets were subjected to the following treatments: 4 °C, 4 °C with 1 µM ABA, 4 °C with 5 µM ABA, 22 °C, 22 °C with 1 µM ABA, and 22 °C with 5 µM ABA. Total RNA was extracted using the Concert™ Plant RNA Reagent Protocol (Invitrogen, Carlsbad, CA, USA). RNA quality was assessed on a 1% agarose gel with 0.5X TBE buffer. Genomic DNA was removed using DNase Turbo (Ambion, Austin, TX, USA). cDNA was then synthesized from 1 μg of total RNA using the SuperScript™ II Reverse Transcriptase kit (Invitrogen, Carlsbad, CA, USA). For *CIR1* gene PCR amplification, 35 cycles were performed with 10 s of denaturation at 95 °C, 30 s of annealing at 60 °C, and a 1 min extension at 72 °C. For the *AtUBQ* housekeeping gene, PCR was performed with 30 cycles, including 10 s of denaturation at 95 °C, 30 s of annealing at 60 °C, and 30 s of extension at 72 °C. Primers are listed in Supplementary Table S2. PCR products were separated on agarose gels: 1.5% for the *CIR1* gene and 1% for the *UBQ5* gene.

### 2.10. Statistical Analysis

To assess statistical significance, one-way ANOVA followed by Tukey’s multiple comparison post-test was conducted, with a significance level set at *p* > 0.05. These statistical analyses were performed using GraphPad Prism version 6.0 (GraphPad Software, CA, USA).

## 3. Results

### 3.1. Differentially Expressed Genes (DEGs) in Arabidopsis thaliana Under Low Temperature Conditions

To identify genes exhibiting differential expression in response to cold stress in the model plant *Arabidopsis thaliana* (Col-0 ecotype), we subjected 14-day-old plantlets to three low temperatures: 0 °C, 4 °C, and 10 °C for a duration of 24 h, along with a control group maintained at 22 °C (Supplementary Figure S1). After 24 h of treatment, the plant material was frozen and prepared for RNA extraction. We analyzed three replicates of each temperature for the sequencing process, which was conducted using the Illumina HiSeq 2500 sequencing platform. Differential transcript expression was assessed by comparing each low-temperature condition to the control condition at 22 °C. After bioinformatic analysis, a total of 38,292 differentially expressed genes (DEGs) were identified through RNA-seq data analysis between each low-temperature condition (0 °C, 4 °C, and 10 °C) and 22 °C with an FDR of <0.01. A total of 23,611 *A. thaliana* transcripts were considered within each contrast analysis. 

**Figure 1:**
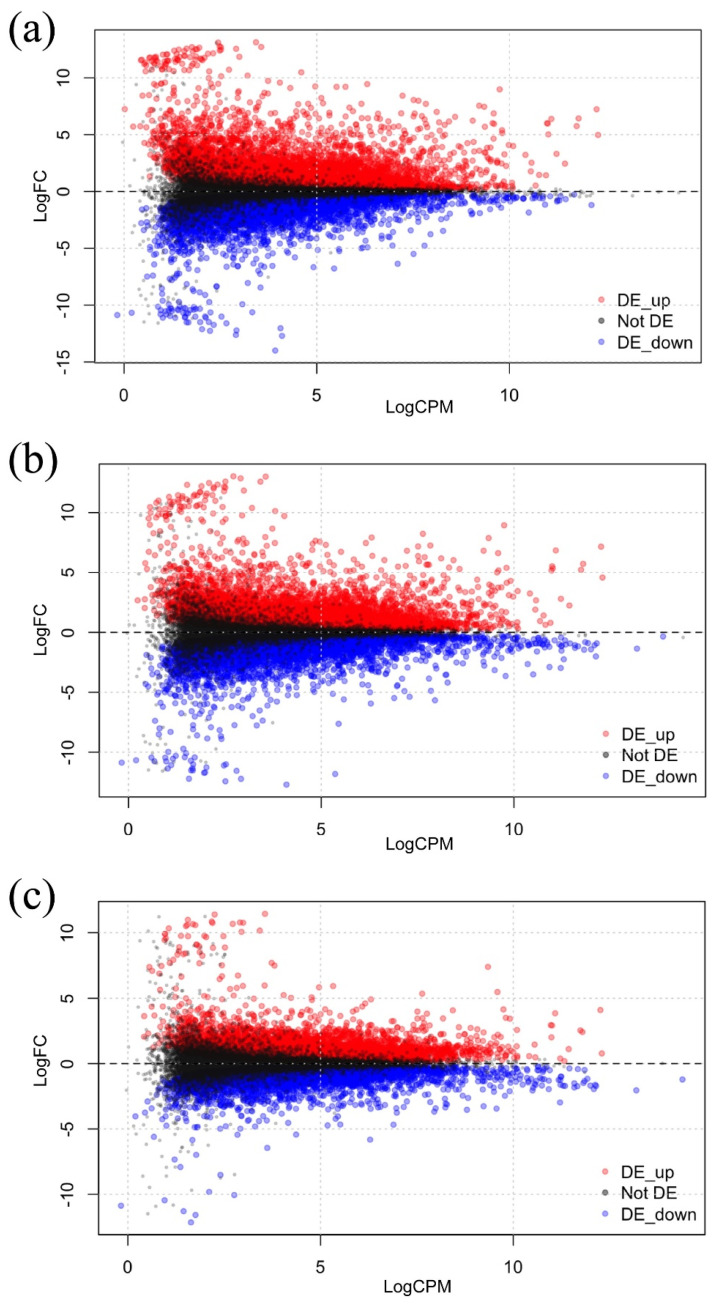
Differentially expressed genes (DEGs) in *Arabidopsis* plantlets exposed to 0 °C, 4 °C, and 10 °C for 24 h, in comparison to the control condition at 22 °C. The plots show the log counts per million (logCPM) on the x-axis, and the log fold change (logFC) of each gene on the y-axis for (**a**) 0 °C, (**b**) 4 °C and (**c**) 10 °C treatments. Each dot represents a gene. Upregulated genes are shown in red, downregulated genes in blue, and genes without change in black. Total transcripts = 23,611, FDR = 0.01.

In Figure 1, the MA plots display the DEGs for each cold treatment. In these plots, the x-axis represents the log ratio (LogCPM), while the y-axis represents the mean average, represented as logFC. When examining the DEGs at each temperature, including both upregulated and downregulated genes within each treatment, we observed that the 0 °C treatment had the highest count of DEGs at 13,861, followed by the 4 °C treatment at 13,729. In contrast, the 10 °C treatment exhibited the lowest number of DEGs, totaling 10,702 (Table 1). The smallest number of DEGs was observed at the more permissive temperature of 10 °C compared to the lower temperatures (0 °C and 4 °C), indicating that the plant regulates a larger set of genes to adapt to colder temperatures (Table 1). 

### 3.2. The 50 Most Expressed Genes at Each Low Temperature of Arabidopsis thaliana Plantlets

**Figure 2:**
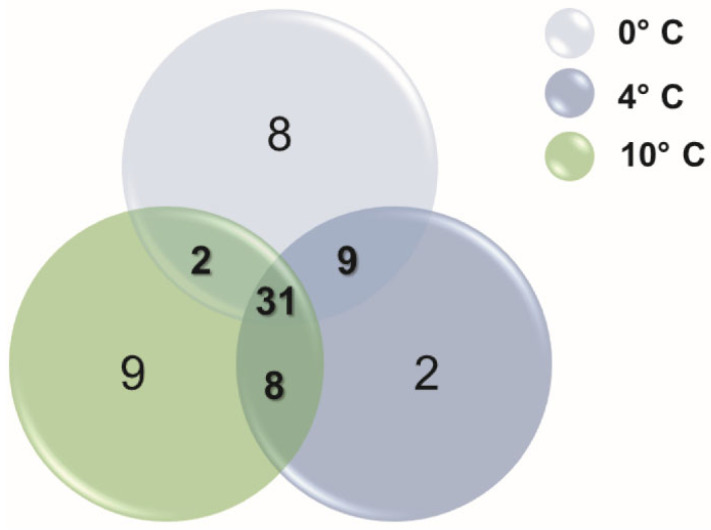
The Venn diagram illustrates the shared upregulated genes among the top 50 genes exhibiting the highest induction across the three cold treatments at 0 °C, 4 °C, and 10 °C.

Using the differentially expressed genes (DEGs) data for each low-temperature condition, we conducted an analysis to identify the top 50 genes with the highest induction in *A. thaliana* plantlets based on their LogFC, with thresholds of FDR < 0.01 and logCPM > 4 for each temperature (0 °C, 4 °C, and 10 °C) relative to the 22 °C control condition (Supplementary Table S3–S5). These thresholds were chosen to ensure the selection of highly expressed genes. We found 31 genes common to *Arabidopsis* plantlets under all three low temperatures by comparing the top 50 cold-induced genes from each temperature (Figure 2). Nine genes were unique to the 0 °C and 4 °C treatments, eight genes to the 4 °C and 10 °C treatments, and only two genes shared between the 0 °C and 10 °C (Figure 2). These findings indicate that a smaller temperature difference, such as between 0 °C/4 °C or 4 °C/10 °C, resulted in a higher number of shared genes compared to the more significant gradient of 0 °C/10 °C.

Table 2 shows the 31 genes shared at the three low temperatures (see Figure 2), listed in descending order based on the LogFC values obtained from the 0 °C treatment. Within these 31 genes, we identified some genes previously characterized as cold response genes, such as *COR15a* and *AtGLR3.4* [31,32,33,34], also genes that code for proteins related to other abiotic stress such as *P5CS2* (salt stress) and *SUS1* (hypoxia and osmotic stress) [35,36,37]. We also identified genes that encode for transcription factors (TFs), such as *HYH* (At3g17609), which encodes a basic leucine zipper (bZIP) transcription factor [38,39], and *CIR1* (At5g37260), which encodes an MYB transcription factor [40]. The rest of the list contains a broader range of genes, including those related to RNA processing, such as binding and editing (*PPR596*) [41], and alternative splicing (*SPF30* and *PRP4KA*) [42,43], among others (Table 2).

Among the nine cold-induced genes shared between the 0 °C and 4 °C treatments (Table 3, Figure 2), we identified *Galactinol synthase 3* (*GolS3*) and *Cold-regulated gene 28* (*COR28*), which have been previously reported as cold-responsive genes in *Arabidopsis* [44]. In addition, there are genes related to the circadian rhythm, such as *COR28*, *TOC1,* and *JMJD5* [45,46,47].

In Table 3, we also showed the eight cold-induced genes exclusively shared between the temperatures of 4 °C and 10 °C. These genes include a PHD-type transcription factor with transmembrane domains (*PTM*), which mediates chloroplast signals to the nucleus [48], genes encoding proteins related to gluconeogenesis (*FBA5*), photosynthesis (*RBCS3B* and *KEA3*) [49,50], and a responsive protein to light and cold stress (*ELIP2*) [51], among others. At 0 °C and 10 °C (Table 3), two shared cold-induced genes were identified: *FAB1D* encodes a 1-phosphatidylinositol-3-phosphate 5-kinase, which is involved in producing phosphatidylinositol (3,5)-bisphosphate and is associated with pH control in the vacuole and endomembrane trafficking [52]; and *MUR4*, encoding UDP-arabinose 4-epimerase 1, a key component in UDP-Arabinose biosynthesis in *Arabidopsis* [53].

### 3.3. Gene Expression Analysis of Selected Genes from RNAseq Data at Low Temperatures (0 °C, 4 °C, and 10 °C) for 24 h, 48 h, and 72 h

**Figure 3:**
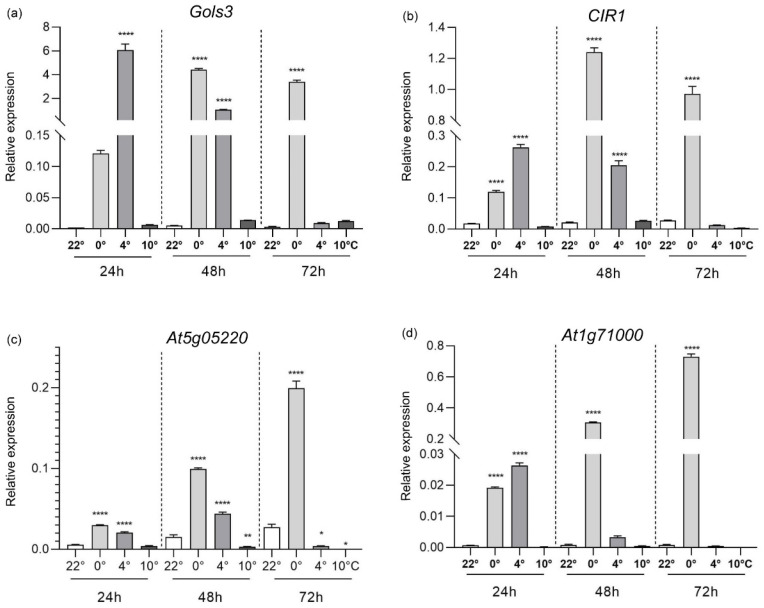
Expression of the *Gols3*, *CIR1*, At5g05220, and At1g71000 genes was evaluated under low temperatures (0 °C, 4 °C, 10 °C), and their respective control at 22 °C, for 24 h, 48 h, and 72 h. Relative gene expression of (**a**) *Gols3*, (**b**) *CIR1*, (**c**) At5g05220, and (**d**) At1g71000 by qRT-PCR analysis. *Arabidopsis thaliana* 14-day-old plantlets were subjected to 0 °C, 4 °C and 10 °C for 24 h, 48 h, and 72 h. Gene expression was measured by qRT-PCR, the values of the relative expression (2^−ΔCt^) were calculated to the reference gene *AtUBQ5*. The mean ± SD of three biological replicates is presented. Asterisks indicate significant differences by one-way ANOVA (*—*p* ≤ 0.05, **—*p* ≤ 0.01 and ****—*p* ≤ 0.0001).

Based on the RNAseq data obtained from the cold treatments (DEGs, induced genes), we selected four genes: *Galactinol synthase 3* (*Gols3*, At1g09350), *CIR1* (At5g37260), *DNAJ* (At1g71000), and At5g05220, to analyze their expression using qRT-PCR (Figure 3). To perform the analysis, 14-day-old *Arabidopsis* plantlets were exposed to temperatures of 0 °C, 4 °C, and 10 °C for duration of 24, 48, and 72 h. Gols3 plays a role in the accumulation of galactinol and raffinose under cold conditions in *Arabidopsis* [54]. We observed that the *Gols3* gene exhibited the highest expression at 24 h and 48 h when exposed to temperatures of 0 °C and 4 °C, while its expression at 72 h was mainly induced at 0 °C (Figure 3a). We examined the *CIR1* gene, which encodes a MYB transcription factor involved in regulating the circadian cycle [40]. The *CIR1* gene showed induction at all three time points at 0 °C, with the highest expression at 48 h, and at 4 °C, it exhibited upregulation at 24 h and 48 h (Figure 3b). Based on transcriptomic data from cold treatments, a splice variant of the *CIR1* gene, which retains intron 3 (*CIR1*-I3R), was identified (see Supplementary Figure S2). We analyzed the expression of *CIR1* and its *CIR1*-I3R version in 14-day-old plants under cold stress (4 °C) and control conditions (22 °C). In addition to temperature treatment, we applied ABA to assess whether the combination of 4 °C and ABA induces expression of the alternative splicing isoform. The following treatments were analyzed by RT-PCR: 4 °C, 4 °C-1 µM ABA, 4 °C-5 µM ABA, 22 °C, 22 °C-1 µM ABA, and 22 °C-5 µM ABA. Our data showed that both the *CIR1* band (751 bp) and the *CIR1-I3R* variant (882 bp) were amplified under the 4 °C and 4 °C-ABA treatments. In contrast, under the control treatments (22 °C and 22 °C-ABA), only the *CIR1* band (751 bp) was amplified. Interestingly, under the 4 °C and 4 °C-ABA treatments, a third band appeared between the *CIR1* (751 bp) and *CIR1*-I3R (882 bp) bands, which may correspond to another intron retention variant, possibly intron 2 (89 bp) or intron 4 (85 bp) (Supplementary Figure S2).

We performed qRT-PCR analysis on the genes At5g05220 and At1g71000. Previous studies have reported that both genes are induced by H_2_O_2_ [55], and At5g05220 is known to be responsive to ABA [56]. At5g05220 encodes a protein of unknown function. In the 0 °C treatment, the At5g05220 gene showed a higher level of induction compared to the other low temperatures, increasing over time and peaking at 72 h. At 4 °C, the At5g05220 gene exhibited induction at 24 h, reaching its maximum expression at 48 h (Figure 3c). Furthermore, the At1g71000 gene, which encodes a DnaJ-type chaperone, displayed its highest expression at 0 °C, with expression increasing over time. In the 4 °C treatment, induction of the At1g71000 gene was observed only at 24 h, in contrast to the control treatment at 22 °C (Figure 3d).

### 3.4. UDP-Glycosyltransferase Genes in A. thaliana Exhibit Differential Expression Under Low Temperatures

**Figure 4:**
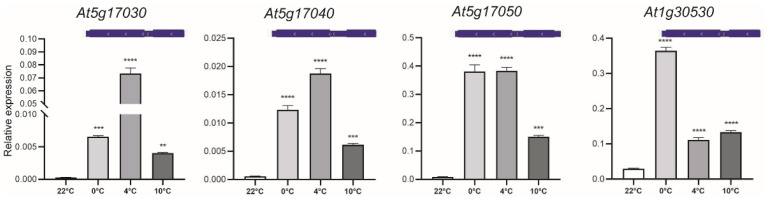
Expression levels of UDP-glucosyltransferase genes induced under low temperature conditions. Scaled representation of UDP-glucosyltransferase genes, including the continuous genes At5g17030, At5g17040, and At5g17050, as well as the At1g30530 gene. Fourteen-day-old *Arabidopsis thaliana* plantlets were exposed to temperatures of 0 °C, 4 °C, and 10 °C for 24 h. Gene expression was measured by qRT-PCR, the values of the relative expression (2^−ΔCt^) were calculated to the reference gene *AtUBQ5*. The mean ± SD of three biological replicates is presented. Asterisks indicate significant differences from the control (**—*p* ≤ 0.01, ***—*p* ≤ 0.001 and ****—*p* ≤ 0.0001), one-way ANOVA.

Within the top 50 most induced genes shared among the three temperatures, we identified the At5g17030 gene, which encodes UDP-glycosyltransferase UGT78D3 (Table 2). Our data indicate that the *UGT78D3* gene was upregulated after 24 h of exposure at 0 °C, 4 °C, and 10 °C, with the highest level of expression observed at 4 °C, in contrast to the control temperature of 22 °C (Figure 4). In addition, we analyzed the other genes belonging to a UGT78 family, including At5g17040 (*UGT78D4*), At5g17050 (*UGT78D2*), and At1g30530 (*UGT78D1*). The contiguous paralogs of At5g17030 (*UGT78D3*), including the genes At5g17040 (*UGT78D4*) and At5g17050 (*UGT78D2*), exhibited increased expression levels at all three lower temperatures, with the most pronounced upregulation occurring at 0 °C and 4 °C when compared to the 22 °C treatment (Figure 4). As for the other paralog, At1g30530 (*UGT78D1*), located on chromosome 1, showed its highest expression at 0 °C, followed by the 4 °C and 10 °C treatments (Figure 4). Our data revealed that all four genes within the UGT78D family were upregulated at all three temperatures, suggesting their potential involvement in the response to cold stress.

### 3.5. Enrichment Analysis of Gene Ontology Induced by Cold Stress

**Figure 5:**
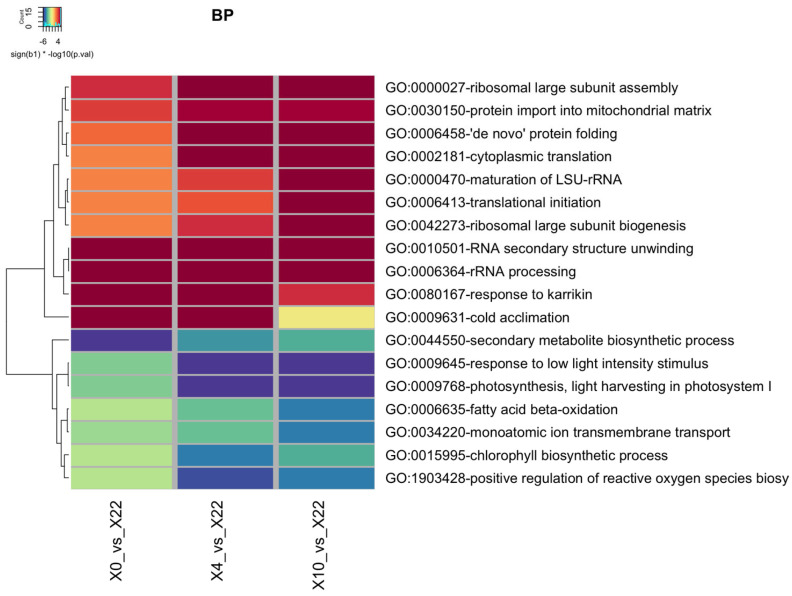
Gene ontology enrichment analysis of genes modulated by cold stress. The heatmap illustrates the main biological processes (BPs) that were upregulated (in various shades of red and orange), downregulated (in various shades of blue and green), and unchanged (in yellow) when 14-day-old plantlets were exposed to low temperatures (0 °C, 4 °C, and 10 °C) compared to the control temperature of 22 °C. The *p*-values and the sign of the enrichment are represented as the scale color in the heatmap (color = sign(enrichment) · −log10 (*p*-value)).

An analysis of gene ontology enrichment was performed on the RNA-seq data obtained during exposure of *Arabidopsis* to cold stress. The objective was to identify and analyze biological processes (BP) enriched commonly across the three specific low-temperature treatments. Figure 5 shows a heatmap representing the main upregulated biological processes (in red/orange shades), downregulated biological processes (in blue/green shades), and processes with no change (in yellow). Complete heatmaps of biological processes, cellular components, and molecular functions are available in Supplementary Figure S3. We observed a trend where the enrichment of BPs, both upregulated and downregulated, showed greater similarity between the 4 °C and 10 °C treatments, with the most significant differences seen in the 0 °C treatment.

Following this, we selected the top five BP categories that showed the highest levels of gene induction (Table 4) or repression (Table 5) within each of the low-temperature treatments. Supplementary Figure S4 shows the 20 BPs with the greatest induction and repression in each cold treatment. Regarding upregulated gene-associated BP, two of the five categories of processes were consistently observed across the three cold treatments; these included “RNA secondary structure unwinding” and “rRNA processing” (Table 4, shown in blue). The processes such as “cold acclimation” and “response to karrikin” were exclusively identified within the top 5 BPs at the coldest temperatures (0 °C and 4 °C, displayed in purple), while “cytoplasmic translation” was present at both 4 °C and 10 °C (shown in green). The remaining BPs were unique to each respective cold treatment (colorless).

**Figure 6:**
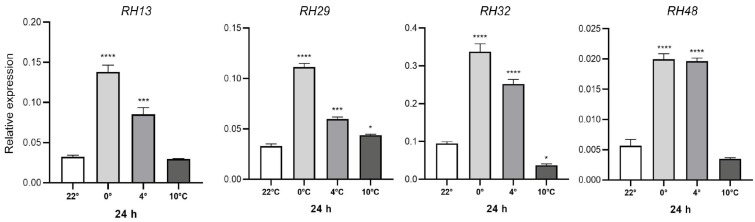
Expression of RNA helicases genes under cold treatments. Relative expression of *RH13*, *RH29*, *RH32*, and *RH48* genes by qRT-PCR at 0 °C, 4 °C and 10 °C for 24 h. *Arabidopsis thaliana* 14-day-old plantlets were subjected to 0 °C, 4 °C and 10 °C for 24 h. Gene expression was measured by qRT-PCR, the values of the relative expression (2^−ΔCt^) were calculated for the reference gene *AtUBQ5*. The mean ± SD of three biological replicates is presented. Asterisks indicate significant differences by one-way ANOVA (*—*p* ≤ 0.05, ***—*p* ≤ 0.001 and ****—*p* ≤ 0.0001).

When examining the category “RNA secondary structure unwinding”, which exhibited enrichment across all three temperatures, we noticed that it consisted of 22 upregulated genes, with 18 of them encoding helicases (Supplementary Table S6). Consequently, we selected to analyze the expression of four RNA helicases, specifically *RH13, RH48, RH32,* and *RH29*, using the qRT-PCR technique. We conducted this analysis in 14-day-old *Arabidopsis* plantlets grown at 0 °C, 4 °C, and 10 °C for 24 h (Figure 6). The four genes encoding RNA helicases displayed increased expression at the lower temperatures of 0 °C and 4 °C when compared to their respective controls at 22 °C. At 10 °C, only the RH29 gene showed a slight increase in expression compared to the 22 °C control (Figure 6). Based on our data, it is suggested that these biological processes, specifically the RNA helicases involved in unwinding RNA secondary structures, could potentially play a role in responding to cold stress.

Through the analysis of the top five BP categories where cold-repressed genes were clustered (Table 5), we identified categories such as “photosynthesis, light harvesting in photosystem I” and “response to low light intensity stimulus”, which were present at all three low temperatures (shown in blue). The category “protein-chromophore binding” was observed as a shared BP between 4 °C and 10 °C (shown in green), while the remaining BPs were specific to individual cold treatments (colorless). The “protein-chromophore linkage” category was observed as a shared BP between 4 °C and 10 °C, whereas the remaining BPs were specific to individual cold treatments (Table 5 and Supplementary Figure S4).

## 4. Discussion

As sessile organisms, plants modulate their expression profiles to generate proteins and compounds that confer tolerance to abiotic stress conditions, including cold. Therefore, understanding transcriptional regulation is crucial for deciphering the plant cell mechanisms used to induce abiotic stress tolerance. In this study, our aim was to identify genes that exhibit differential expression in *Arabidopsis thaliana* plantlets when exposed to low temperatures for 24 h. While *A. thaliana* is typically a temperate plant, our study revealed that the expression of over one-fifth of the poly(A) transcriptome was modulated when plantlets were exposed to low temperatures for a short period. Specifically, the colder temperatures (0 °C and 4 °C) resulted in the upregulation and downregulation of over 6600 genes in comparison to plantlets grown at 22 °C. Moreover, the milder cold treatment of 10 °C, which represents an intermediate temperature between the optimal temperature of 22 °C and 0 °C, also exhibited significant regulation of genes, with 5514 genes being upregulated and 5188 genes being downregulated.

We selected and quantified the expression levels of genes, including *Gols3* and *CIR1*, based on RNA-seq analysis. Increased expression of *Gols3* and *CIR1* was observed at all three analyzed time points (24 h, 48 h, and 72 h) when exposed to 0 °C. Similarly, under the 4 °C treatment, both genes exhibited upregulation at 24 h and 48 h. Therefore, the *Gols3* and *CIR1* genes could play an important role in responding to cold stress, especially at temperatures as low as 4 °C and 0 °C. The *CIR1* gene (also known as *RVE2*) encodes an MYB transcription factor that plays a role in regulating the circadian cycle, and acts as a regulator for the expression of cold response genes and cold tolerance [40]. The loss-of-function of *Arabidopsis CIR1* reduces freezing tolerance, whereas *CIR1* overexpression lines showed increased tolerance to freezing before and after cold acclimation [34]. We identified an alternative splice variant of the *CIR1* gene that retains intron 3 (*CIR1*-I3R). *CIR1* and its variant (*CIR1*-I3R) were expressed at 4 °C, both with and without the presence of two concentrations of ABA. However, *CIR1*-I3R was not detected without cold treatment at 22 °C, even with the application of ABA. The *CIR1*-I3R variant, which retains intron 3 of the *CIR1* gene, includes a premature stop codon. This results in a truncated CIR1 protein consisting of 78 amino acids, comprising the first 62 amino acids of the CIR1 protein and an additional 16-amino acid sequence encoded within intron 3. We also detected a third band under cold stress, specifically at 4 °C with and without ABA treatments, which likely corresponds to another alternative splicing variant, where either intron 2 or intron 4 is retained (Supplementary Figure S2). The alternative splicing variants of the *CIR1*/*RVE2* gene have been previously reported [57,58]. James et al. (2023) report that, besides the full-length version encoding the CIR1/RVE2 transcription factor with the MYB domain (287 amino acids), six alternatively spliced forms have been identified. These forms are likely nonfunctional, as they produce transcripts with a premature termination codon either after 4 or 23 amino acids, before the MYB domain. In this study, the *CIR1*-I3R variant encodes a predicted peptide of 78 amino acids, containing only part of the MYB domain (32 of the 55 amino acids), making it unlikely to function as an active transcription factor. Additional studies will be needed to elucidate the mechanisms underlying *CIR1*/*RVE2* alternative splicing variants and their role in cold stress.

The *A. thaliana Gols3* gene encodes a key enzyme in the raffinose biosynthetic pathway [44]. Klotke et al. (2004) reported a correlation between raffinose accumulation and cold tolerance in *Arabidopsis*, with higher levels of raffinose observed in the cold-tolerant accession (Col-0) compared to the cold-sensitive accession (C24) [59]. The synthesis of metabolites has been documented as a crucial factor in the response of plants to cold stress. These cold-responsive metabolites, including amino acids, betaines, soluble sugars, organic acids, polyols, lipids, and polyamines, exhibit cryoprotective and scavenging activities, while also serving as stabilizers for proteins and enzymes, as well as regulators of gene expression [6].

Interestingly, we identified for the first time two genes that are induced under cold stress, such as the DnaJ chaperone (At1g71000) and At5g05220. Although the At5g05220 gene is of unknown function, it has been reported that the At5g05220 gene encodes an H_2_O_2_-induced protein, which was localized in chloroplasts [56]. Our data show that the At5g05220 gene was upregulated at cooler temperatures, 4 °C and 0 °C. Further studies will be necessary to elucidate the function of the At5g05220 gene under cold stress. The At1g71000 gene encodes a DnaJ chaperone (HSP40), which collaborates with HSP70 to facilitate protein folding, refolding, and degradation [60]. When analyzing the expression of the At1g71000 gene, we observed significant induction at 0 °C across the three analyzed time points (24 h, 48 h, and 72 h). Notably, at 4 °C, expression was observed only after 24 h of cold exposure. The transient expression of the At1g71000 gene in *Arabidopsis* mesophyll protoplasts showed nucleo-cytosolic localization of the DNAJ protein. Subsequent to the addition of 1.5 mM H_2_O_2_, an exclusively nuclear localization was observed in approximately 15% of the protoplasts [56], suggesting a potential role for this DnaJ in abiotic stress responses. Tomato SlDnaJ20, a chloroplast-localized DnaJ protein, is upregulated in response to cold stress. Overexpressing SlDnaJ20 in tomatoes mitigated cold-induced damage to PSI and PSII complexes, enhancing their stability and, consequently, improving transgenic tomato tolerance to cold stress [61]. All the evidence above suggests that DnaJ functions as a molecular chaperone to protect plant proteins from low temperatures.

Within the top 50 induced genes and shared in the three cold treatments, we found the At5g17030 gene, which codes for the UDP-glucosyltransferase (UGT). We analyzed the expression of the At5g17030 (UGT78D3) gene under cold conditions, along with its contiguous paralogs At5g17040 (UGT78D4) and At5g17050 (UGT78D2), which belong to the same UGT family. Additionally, we examined a fourth member, At1g30530 (UGT78D1), located on chromosome 1. Based on our data, it was observed that four genes belonging to the UGT78D family exhibited upregulation at all three temperatures after 24 h, indicating their potential role in the cold stress response. The UDP-glycosyltransferases facilitate the transfer of glycosyl residues to acceptor molecules, thereby regulating the properties of the acceptors, including bioactivity, solubility, and transport [62]. In *A. thaliana*, UGT79B2 and UGT79B3 have been reported to enhance plant tolerance to low temperatures, as well as drought and salt stresses, by modulating anthocyanin accumulation [63]. Chen et al. (2020) recently reported that *Arabidopsis* UGT75B1 plays a role in regulating the plant’s stress response by modifying ABA glycosylation [64]. The authors propose that UGT75B1 catalyzes the conversion of excess ABA into its inactive glucose ester to prevent the overaccumulation of ABA and buffer its activity.

We conducted a gene ontology enrichment analysis on *Arabidopsis* RNAseq data under cold stress to identify commonly enriched biological processes (BP) across three distinct low-temperature treatments. The BP most upregulated in the three cold treatments were “RNA secondary structure unwinding” and “rRNA processing”, suggesting that the plant cell is expressing genes involved in facilitating mRNA translation and some related to ribosome biogenesis to adapt to cold conditions. Interestingly, upon analyzing the category of ‘RNA secondary structure unwinding’, we observed that the majority of the genes encode helicases. Helicases are a class of enzymes that catalyze the unwinding of energetically stable duplex RNA secondary structures in an ATP-dependent manner [65]. The RNA helicases participate in diverse processes, including remodeling of RNA structures, RNA splicing, translation initiation, ribosome assembly, rRNA processing, and nuclear mRNA transport, among others [13]. The four selected RNA helicases for transcriptional analysis, including *RH13, RH48, RH32,* and *RH29* genes, all exhibited an induction of expression at lower temperatures (0 °C and 4 °C) compared to their respective controls at 22 °C.

The *Arabidopsis Los4* gene, which encodes the DEAD-box RNA helicase (AtRH38), plays a role in cold tolerance by regulating the export of mRNA from the nucleus to the cytoplasm during cold stress conditions [66]. In 2008, Kim et al. reported an increased freezing tolerance in *Arabidopsis* plants through overexpression of the *AtRH25* gene [67]. Furthermore, complementing the *AtRH25* gene in mutant bacteria alleviated the cold-sensitive phenotype of BX04 cells at low temperatures, suggesting a potential role for *AtRH25* as an RNA chaperone during plant cold adaptation. It has been reported that the RH7 helicase plays a role in the response to plant cold stress. RH7 interacts with the chaperone CSP3 (Cold Shock Domain Protein 3) in the nucleolus, regulating the secondary structures of rRNA and ensuring the accurate processing of pre-rRNA [68]. Therefore, the importance of RNA helicases becomes evident in responses to cold stress. At low temperatures, RNA molecules are prone to forming stable secondary structures that impede functionality. RNA helicases play a crucial role in unwinding these structures, thereby ensuring the proper functioning of RNA molecules under cold stress conditions. At the coldest temperatures, such as 0 °C and 4 °C, biological processes such as “cold acclimatization” and “karrikin response” were exclusively identified within the top five BPs. Karrikins are small organic compounds found in burnt or charred plant material and their smoke; they have been reported to stimulate seed germination in various plants [69]. Karrikins have been proposed to contribute to abiotic stress tolerance, especially in the cold response of *Chorispora bungeana* and *Arabidopsis* [70].

Regarding the biological processes that were repressed, “Photosynthesis, light harvesting in photosystem I” and “Response to low light intensity stimulus” were shared in the top 5 in the three temperatures. As widely reported, the photosynthetic apparatus is highly sensitive to abiotic stresses, and cold is no exception. Processes related to photosynthesis and CO_2_ fixation are affected when plants are exposed to low temperatures [71]. Our data reveal that despite the brief 24-h cold treatment applied to *A. thaliana* seedlings, cells are already repressing genes associated with the photosynthetic process.

## 5. Conclusions

Cold stress negatively impacts both the growth and development of plants, causing harm to cells and impeding overall growth. Furthermore, lower temperatures restrict the geographic range of plant species and adversely affect crop productivity. Molecular mechanisms, which encompass the reprogramming of gene expression, are crucial for tolerating abiotic stress. Our data reveal a consistent enrichment of biological processes related to translation across all three low-temperature conditions. This indicates that plant cells express genes facilitating mRNA translation to adapt to low-temperature exposure, including processes such as RNA secondary structure unwinding, rRNA processing, and cytoplasmic translation. This study offers valuable insights into the mechanisms of cold tolerance at three low temperatures in the model plant *A. thaliana*, contributing to future research aimed at enhancing cold tolerance in plants.

## Figures and Tables

**Table 1:** Differentially expressed genes (DEGs) at 0 °C, 4 °C, and 10 °C relative to the control temperature of 22 °C, including both upregulated and downregulated genes for each treatment. Total transcripts = 23,611, FDR = 0.01.

Treatment	Up	Down	Total
0 °C vs. 22 °C	6690	7171	13,861
4 °C vs. 22 °C	6910	6819	13,729
10 °C vs. 22 °C	5514	5188	10,702

**Table 2:** The *Arabidopsis thaliana* genes most induced in the three cold treatments. Genes shared among *A. thaliana* plantlets across all three low temperatures by analyzing the top 50 cold-induced genes from each temperature. The genes are arranged in descending order of expression (LogFC) based on their values obtained at 0 °C. The light gray color indicates the 0 °C treatment, the gray color indicates the 4°C treatment, and the green color indicates the 10 °C treatment.

			LogFC			
**No.**	**Gene ID**	**Protein**	**0 °C**	**4 °C**	**10 °C**	**Description**
1	At3g50380	AT3G50380	13.1	12.6	10.2	Vacuolar protein sorting-associated protein 13b
2	At2g36850	GSL8	12.8	12.2	8.1	Callose synthase 10
3	At3g63460	SEC31B	12.7	13.0	11.4	Protein transport protein SEC31 homolog B
4	At4g00710	BSK3	12.7	12.1	8.3	Serine/threonine-protein kinase BSK3
5	At1g21380	TOL3	12.6	12.9	10.8	TOM1-like protein 3
6	At5g17030	UGT78D3	12.5	12.6	8.3	UDP-glycosyltransferase 78D3
7	At2g02570	SPF30	12.4	11.8	9.7	Survival of motor neuron-related-splicing factor 30
8	At3g54500	LNK2	12.2	12.3	8.4	Night light-inducible and clock-regulated gene 2
9	At5g47430	AT5G47430	12.1	11.5	10.8	E3 ubiquitin ligase PQT3-like
10	At3g55610	P5CS2	11.9	11.7	8.5	Delta-1-pyrroline-5-carboxylate synthase B
11	At4g16990	RLM3	11.9	11.8	10.8	Disease resistance protein RLM3
12	At1g01060	LHY	11.8	12.5	9.1	Late elongated hypocotyl
13	At5g62570	CBP60A	11.7	11.0	9.8	Calmodulin-binding protein 60 A
14	At4g25450	ABCB28	11.6	11.4	11.2	ABC transporter B family member 28
15	At2g42540	COR15A	11.6	11.7	9.0	Protein COLD-REGULATED 15A
16	At5g37130	AT5G37130	11.6	11.2	6.7	Tetratricopeptide repeat protein 27 homolog
17	At4g36980	AT4G36980	11.5	10.9	5.4	CLK4-associating serine/arginine rich protein
18	At2g23420	NAPRT2	11.1	12.2	8.5	Nicotinate phosphoribosyltransferase 2
19	At3g57660	NRPA1	11.1	10.5	10.6	DNA-directed RNA polymerase I subunit 1
20	At3g17609	HYH	10.7	13.0	10.7	Transcription factor HY5-like
21	At1g80270	PPR596	10.7	11.4	10.6	Pentatricopeptide repeat-containing protein
22	At5g20830	SUS1	10.6	12.0	8.8	Sucrose synthase 1
23	At3g25840	PRP4KA	10.2	9.5	8.9	Serine/threonine-protein kinase PRP4 homolog
24	At1g11720	SS3	10.0	9.9	10.7	Starch synthase 3
25	At1g05200	GLR3.4	9.5	11.0	11.4	Glutamate receptor 3.4
26	At4g01985	AT4G01985	9.5	9.2	6.7	Uncharacterized protein
27	At5g37260	CIR1	9.3	7.6	5.8	MYB family transcription factor Circadian 1
28	At1g48540	AT1G48540	9.1	9.3	8.2	Outer arm dynein light chain 1, protein kinase binding
29	At1g03080	NET1D	8.6	8.7	7.7	Protein NETWORKED 1D
30	At4g31210	AT4G31210	8.3	9.8	10.1	DNA topoisomerase 1
31	At5g52310	LTI78	8.0	8.2	5.3	Low-temperature-induced 78 kDa protein

**Table 3:** Genes most induced in *Arabidopsis thaliana* between 0–4 °C, 4–10 °C, and 0–10 °C treatments. These genes are shared among *A. thaliana* plantlets across two low temperatures, selected from the top 50 cold-induced genes at each temperature, and are arranged in descending order of expression (LogFC) based on their values obtained at 0 °C or 4 °C. The light gray color indicates the 0 °C treatment, the gray color indicates the 4°C treatment, and the green color indicates the 10 °C treatment.

			LogFC		
**No.**	**Gene ID**	**Protein**	**0 °C**	**4 °C**	**Description**
1	At2g24560	GGL15	10.2	10.4	Guard cell-enriched GDSL lipase 15
2	At5g61380	TOC1	9.5	7.9	Timing of Cab expression 1
3	At1g72440	EDA25	9.2	8.3	Embryo sac development arrest 25
4	At1g09350	Gols3	9.0	8.9	Galactinol synthase 3
5	At3g20810	JMJD5	8.8	7.8	Lysine-specific demethylase JMJ30
6	At4g33980	COR28	8.7	7.9	Cold-regulated gene 28
7	At4g18422	At4g18422	8.2	7.3	Transmembrane protein
8	At2g42520	RH37	7.7	7.4	RNA Helicase 37
9	At1g70640	At1g70640	7.7	7.5	Octicosapeptide/Phox/Bem1p (PB1) domain-containing protein
**No.**	**ID Gene**	**Protein**	**4 °C**	**10 °C**	**Description**
1	At3g51240	F3H	10.1	7.5	Flavanone 3-hydroxylase
2	At4g26530	FBA5	9.5	10.6	Fructose-bisphosphate aldolase 5
3	At5g38410	RBCS3B	8.5	8.3	Ribulose bisphosphate carboxylase small chain 3B
4	At4g14690	ELIP2	7.9	7.4	Early light-induced protein 2
5	At3g63340	AT3G63340	7.7	6.7	Probable protein phosphatase 2C 51
6	At5g35210	PTM	7.6	5.8	PHD type TF with transmembrane domains
7	At5g27970	At5g27970	7.4	6.5	ARM repeat superfamily protein
8	At4g04850	KEA3	7.4	5.9	K(+) efflux antiporter 3
**No.**	**ID Gene**	**Protein**	**0 °C**	**10 °C**	**Description**
1	At1g34260	FAB1D	8.5	5.6	Putative 1-phosphatidylinositol-3-phosphate 5-kinase
2	At1g30620	MUR4	8.4	5.6	UDP-arabinose 4-epimerase 1

**Table 4:** The top five categories of biological processes (BPs) exhibiting the highest levels of gene induction (positive enrichment) at 0 °C, 4 °C, and 10 °C compared to the control temperature of 22 °C. The BPs shared among the three temperatures analyzed are shown in blue; those shared exclusively between the 0 °C and 4 °C treatments are in purple, and those shared between 4°C and 10 °C are in green.

	Positively Enriched Biological Processes		
**No.**	**0 °C**	**4 °C**	**10 °C**
1	RNA secondary structure unwinding	RNA secondary structure unwinding	Cytoplasmic translation
2	rRNA processing	rRNA processing	Ribosomal large subunit assembly
3	Cold acclimation	Response to karrikin	rRNA processing
4	Response to karrikin	Cytoplasmic translation	RNA secondary structure unwinding
5	Response to UV-B	Cold acclimation	Ribosomal small subunit assembly

**Table 5:** The top five biological process (BP) categories with the lowest levels of gene expression (negative enrichment) at 0 °C, 4 °C, and 10 °C compared to the control temperature of 22 °C. The BPs shared among the three analyzed temperatures are shown in blue, whereas those shared exclusively between 4 °C and 10 °C are shown in green.

Negatively Enriched Biological Processes			
**No.**	**0 °C**	**4 °C**	**10 °C**
1	Secondary metabolite biosynthetic process	Photosynthesis, light harvesting in photosystem I	Protein-chromophore linkage
2	Pectin catabolic process	Protein-chromophore linkage	Photosynthesis, light harvesting in photosystem I
3	Plant-type cell wall organization	Response to low light intensity stimulus	Response to low light intensity stimulus
4	Photosynthesis, light harvesting in photosystem I	Reductive pentose-phosphate cycle	Autophagy
5	Response to low light intensity stimulus	Photorespiration	Response to far red light

## Data Availability

The original contributions presented in the study are included in the article/Supplementary Material, and original RNA-seq data is openly available in Gene Expression Omnibus under accession GSE27881.

## Supplementary Materials

The following supporting information can be downloaded at: https://www.mdpi.com/article/10.3390/plants13223127/s1, Table S1. RNA-seq mapping statistics. Table S2. List of primers used in this study. Table S3. The top 50 cold-induced genes in *Arabidopsis thaliana* at 0 °C treatment, determined by their LogFC values relative to the 22 °C control condition. Table S4. The top 50 cold-induced genes in *Arabidopsis thaliana* at 4 °C treatment, determined by their LogFC values relative to the 22 °C control condition. Table S5. The top 50 cold-induced genes in *Arabidopsis thaliana* at 10 °C treatment, determined by their LogFC values relative to the 22 °C control condition. Table S6. Genes in the ‘RNA secondary structure unwinding’ category of upregulated biological processes (BPs). Figure S1. *Arabidopsis thaliana* plantlets exposed to cold stress. Representative plates of 14-day-old plantlets after being exposed to low temperatures (0 °C, 4 °C, and 10 °C), and a control temperature (22 °C), for 24 h. Figure S2. Genomic organization of the *CIR1* gene and its alternatively spliced version *CIR1-I3R*, along with their amplification by RT-PCR. Figure S3. Gene ontology enrichment analysis of genes regulated by cold stress. Figure S4. Enriched biological processes (BP) are indicated in red (upregulated) and blue (downregulated) at temperatures (a) 0 °C, (b) 4 °C, and (c) 10 °C.
