# Supplementary material for: Transcriptome Analysis Reveals Genes Responsive to Three Low-Temperature Treatments in Arabidopsis thaliana
Source: Plants (Basel). 2024 Nov 6;13(22):3127. doi: 10.3390/plants13223127 (PMC11597575; doi:10.3390/plants13223127)
Supplement: Supplementary file 1 [file plants-13-03127-s001.zip › plants-3236391-supplementary.pdf]

Transcriptome analysis reveals genes responsive to three low-temperature treatments in *Arabidopsis thaliana*

Bricia Ruiz-Aguilar[1], Natalia Torres-Serrallonga[1], María Azucena Ortega-Amaro[1,2], Arianna Duque-Ortiz[1], Cesaré Ovando-Vázquez[3], and Juan Francisco Jiménez-Bremont[1*]

[1] Laboratorio de Biotecnología Molecular de Plantas, División de Biología Molecular, Instituto Potosino de Investigación Científica y Tecnológica A. C., San Luis Potosí, S.L.P., México.

[2] Coordinación Académica Región Altiplano Oeste, Universidad Autónoma de San Luis Potosí, Salinas de Hidalgo, S.L.P., México.

[3] CONACyT–Centro Nacional de Supercómputo, Instituto Potosino de Investigación Científica y Tecnológica, A.C., San Luis Potosí, S.L.P., México.

[*] Corresponding author: jbremont@ipicyt.edu.mx

Supplementary Table S1. RNA-seq mapping statistics

Library	Mapped reads	Mapped Prop (%)	Unmapped reads	Unmapped Prop (%)
0-R1-2_S4	31497606.5	93.5	2173460.5	6.5
0-R2-2_S5	33544842	93.7	2244761	6.3
0-R3-2_S6	33462772	93.9	2178659	6.1
4-R1-2_S7	40071994.5	93.8	2642571.5	6.2
4-R2-2_S8	31991333.5	93.9	2080447.5	6.1
4-R3-2_S9	34124176	94.0	2193480	6.0
10-R1-2_S10	36355604	94.1	2293540	5.9
10-R2-2_S11	34066842.5	93.9	2198983.5	6.1
10-R3-2_S12	32234052.5	93.6	2186488.5	6.4
22-R1-2_S1	32033617.5	93.7	2166280.5	6.3
22-R2-2_S2	38717653.5	94.0	2482456.5	6.0
22-R3-2_S3	33036667	94.3	2013006	5.7

Supplementary Table S2. List of primers used in this study.

Gene ID	Protein	Primer direction	Primer sequence (5´ to 3´)
At1g09350	GOLS3	Fw	GTGCCAAAGCTCCATCCGC
		Rv	CAATTTCCTAAGTAAACATCACCAG
At5g05220	At5g05220	Fw	CATCAAAGAACAGAGAGAGCAGTC
		Rv	GAGGTACAACGGTACAAGGAATC
At1g71000	DnaJ	Fw	CACAGATGTCTCTACCGCTTTC
		Rv	CACAGTCCGAGTCTCCTAGAG
At5g17030	UGT78D3	Fw	AAGAACTAGCACAAGAAGCTGTCT
		Rv	CACAATGCAAGATAACAACTAGTA
At5g17040	At5G17040	Fw	CTTTGAGAATTTCAAAGGTTTG
		Rv	AAGGTCTTTTCATTTTCATTCCAA
At5g17050	UGT78D2	Fw	GATGGTAAGAAGATGAAATGTAAT
		Rv	AGAAACTGTTGTTATTTATTTGTCCA
At1g30530	UGT78D1	Fw	CATAGTCAAATAAGTGTGTGCCTA
		Rv	CATAGATCCGAGTAAATATAGATATC
At5g37260	CIR1 (qRTPCR)	Fw	CTGAAGATGGCAAGAAGAAGCTATACTCAG
		Rv	GAACCCTCTCATGTTGTTCATCTCAGTC
At5g37260	CIR1 (semi-RTPCR)	Fw	ACTTATATCTTCCTCAGATGCCTT
		Rv	GAACCCTCTCATGTTGTTCATCTCAGTC
At1g63250	RH48	Fw	TTGGTACTTAGATGTATCGTCGTAA
		Rv	AACAAACTTCCTCAAGTAGTCAAAT
At5g54910	RH32	Fw	ATGGCTCTCAAATTCATAACG
		Rv	TTCCTCATAGTAAGACGGTAACAA
At3g16840	RH13	Fw	ATGAAAGGTCAGAGTGCAGAG
		Rv	TAATTAAGAAGAAGCCATTAGTTTTCTC
At1g77030	RH29	Fw	GGAAGATAGTCATGAAGAAGAAGAC
		Rv	AACAAGATTATAAAGTGTTGCACAA

Supplementary Table S3. The top 50 cold-induced genes in *Arabidopsis thaliana* at 0°C treatment, determined by their LogFC values relative to the 22°C control condition.

No.	Gene ID	Protein	LogFC	Description
1	At3g50380	AT3G50380	13.1	Vacuolar protein sorting-associated protein 13b
2	At2g36850	GSL8	12.8	Callose synthase 10
3	At3g63460	SEC31B	12.7	Protein transport protein SEC31 homolog B
4	At4g00710	BSK3	12.7	Serine/threonine-protein kinase BSK3
5	At1g21380	TOL3	12.6	TOM1-like protein 3
6	At5g17030	UGT78D3	12.5	UDP-glycosyltransferase 78D3
7	At2g02570	SPF30	12.4	Survival of motor neuron-related-splicing factor 30
8	At3g54500	LNK2	12.2	Night light-inducible and clock-regulated gene 2
9	At5g47430	AT5G47430	12.1	E3 ubiquitin ligase PQT3-like
10	At3g55610	P5CS2	11.9	Delta-1-pyrroline-5-carboxylate synthase B
11	At4g16990	RLM3	11.9	Disease resistance protein RLM3
12	At1g01060	LHY	11.8	Late elongated hypocotyl
13	At5g62570	CBP60A	11.7	Calmodulin-binding protein 60 A
14	At4g25450	ABCB28	11.6	ABC transporter B family member 28
15	At2g42540	COR15A	11.6	Protein COLD-REGULATED 15A
16	At5g37130	AT5G37130	11.6	Tetratricopeptide repeat protein 27 homolog
17	At4g36980	AT4G36980	11.5	CLK4-associating serine/arginine rich protein
18	At2g23420	NAPRT2	11.1	Nicotinate phosphoribosyltransferase 2
19	At3g57660	NRPA1	11.1	DNA-directed RNA polymerase I subunit 1
20	At3g17609	HYH	10.7	Transcription factor HY5-like
21	At1g80270	PPR596	10.7	Pentatricopeptide repeat-containing protein
22	At5g20830	SUS1	10.6	Sucrose synthase 1
23	At2g24560	GGL15	10.2	Guard cell-enriched GDSL lipase 15
24	At3g25840	PRP4KA	10.2	Serine/threonine-protein kinase PRP4 homolog
25	At1g11720	SS3	10.0	Starch synthase 3
26	At1g05200	GLR3.4	9.5	Glutamate receptor 3.4
27	At4g01985	AT4G01985	9.5	Uncharacterized protein
28	At5g61380	TOC1	9.5	Timing of Cab expression 1
29	At5g37260	RVE2	9.3	MYB family transcription factor Circadian 1
30	At1g71000	AT1G71000	9.2	Chaperone protein DnaJ
31	At1g72440	EDA25	9.2	CCAAT/enhancer-binding protein zeta
32	At3g61190	BAP1	9.1	BON1-associated protein 1

33	At1g48540	AT1G48540	9.1	Outer arm dynein light chain 1, protein kinase binding
34	At1g09350	GOLS3	9.0	Galactinol synthase 3
35	At3g20810	JMJD5	8.8	Lysine-specific demethylase
36	At4g33980	COR28	8.7	Cold regulated gene 28
37	At1g26790	CDF6	8.7	Dof zinc finger protein DOF1.3
38	At1g03080	NET1D	8.6	Protein NETWORKED 1D
39	At1g34260	FAB1D	8.5	Putative 1-phosphatidylinositol-3-phosphate 5-kinase
40	At5g06760	LEA4-5	8.4	Late embryogenesis abundant protein 46
41	At1g51090	ATHMAD1	8.4	Heavy metal-associated isoprenylated plant protein 9
42	At1g30620	MUR4	8.4	UDP-arabinose 4-epimerase 1
43	At4g31210	AT4G31210	8.3	DNA topoisomerase 1
44	At4g18422	AT4G18422	8.2	Transmembrane protein
45	At1g04570	AT1G04570	8.1	Probable folate-biopterin transporter 8
46	At5g52310	LTI78	8.0	Low-temperature-induced 78 kDa protein
47	At5g17300	RVE1	7.9	Protein REVEILLE 1
48	At5g12030	HSP17.6A	7.9	17.7 kDa class II heat shock protein
49	At2g42520	RH37	7.7	RNA Helicase 37
50	At1g70640	AT1G70640	7.7	Octicosapeptide/Phox/Bem1p (PB1) domain-containing protein

Supplementary Table S4. The top 50 cold-induced genes in *Arabidopsis thaliana* at 4°C treatment, determined by their LogFC values relative to the 22°C control condition.

No.	Gene ID	Protein	LogFC	Description
1	At3g17609	HYH	13.0	Transcription factor HY5-like
2	At3g63460	SEC31B	13.0	Protein transport protein SEC31 homolog B
3	At1g21380	TOL3	12.9	TOM1-like protein 3
4	At5g17030	UGT78D3	12.6	UDP-glycosyltransferase 78D3
5	At3g50380	AT3G50380	12.6	Vacuolar protein sorting-associated protein 13b
6	At1g01060	LHY	12.5	Late elongated hypocotyl
7	At3g54500	LNK2	12.3	Night light-inducible and clock-regulated gene 2
8	At2g23420	NAPRT2	12.2	Nicotinate phosphoribosyltransferase 2
9	At2g36850	GSL8	12.2	Callose synthase 10
10	At4g00710	BSK3	12.1	Serine/threonine-protein kinase BSK3
11	At5g20830	SUS1	12.0	Sucrose synthase 1
12	At4g16990	RLM3	11.8	Disease resistance protein RLM3
13	At2g02570	SPF30	11.8	Survival of motor neuron-related-splicing factor 30
14	At2g42540	COR15A	11.7	Protein COLD-REGULATED 15A
15	At3g55610	P5CS2	11.7	Delta-1-pyrroline-5-carboxylate synthase B
16	At5g47430	AT5G47430	11.5	E3 ubiquitin ligase PQT3-like
17	At4g25450	ABCB28	11.4	ABC transporter B family member 28
18	At1g80270	PPR596	11.4	Pentatricopeptide repeat-containing protein
19	At5g37130	AT5G37130	11.2	Tetratricopeptide repeat protein 27 homolog
20	At5g62570	CBP60A	11.0	Calmodulin-binding protein 60 A
21	At1g05200	GLR3.4	11.0	Glutamate receptor 3.4
22	At4g36980	AT4G36980	10.9	CLK4-associating serine/arginine rich protein
23	At3g57660	NRPA1	10.5	DNA-directed RNA polymerase I subunit 1
24	At2g24560	GGL15	10.4	Guard cell-enriched GDSL lipase 15
25	At3g51240	F3H	10.1	Flavanone 3-hydroxylase
26	At1g11720	SS3	9.9	Starch synthase 3
27	At4g31210	AT4G31210	9.8	DNA topoisomerase 1
28	At3g25840	PRP4KA	9.5	Serine/threonine-protein kinase PRP4 homolog
29	At4g26530	FBA5	9.5	Fructose-bisphosphate aldolase 5
30	At1g48540	AT1G48540	9.3	Outer arm dynein light chain 1, protein kinase binding
31	At4g01985	AT4G01985	9.2	Uncharacterized protein
32	At1g09350	GOLS3	8.9	Galactinol synthase 3

33	At1g03080	NET1D	8.7	Protein NETWORKED 1D
34	At5g38410	RBCS3B	8.5	Ribulose bisphosphate carboxylase small chain 3B
35	At1g72440	EDA25, SWA2	8.3	CCAAT/enhancer-binding protein zeta
36	At5g52310	LTI78	8.2	Low-temperature-induced 78 kDa protein
37	At5g61380	TOC1	7.9	Timing of Cab expression 1
38	At4g33980	COR28	7.9	Cold regulated gene 28
39	At4g14690	ELIP2	7.9	Early light-induced protein 2
40	At3g20810	JMJD5	7.8	Lysine-specific demethylase
41	At3g63340	AT3G63340	7.7	Probable protein phosphatase 2C 51
42	At4g25433	AT4G25433	7.6	Peptidoglycan-binding LysM domain-containing protein
43	At5g35210	PTM	7.6	PHD type transcription factor with transmembrane domains
44	At5g37260	RVE2	7.6	MYB family transcription factor Circadian 1
45	At1g70640	AT1G70640	7.5	Octicosapeptide/Phox/Bem1p (PB1) domain-containing protein
46	At1g66700	PXMT1	7.5	Paraxanthine methyltransferase 1
47	At5g27970	AT5G27970	7.4	Uncharacterized protein, ARM repeat superfamily protein
48	At2g42520	RH37	7.4	RNA Helicase 37
49	At4g04850	KEA3	7.4	K(+) efflux antiporter 3
50	At4g18422	AT4G18422	7.3	Transmembrane protein

Supplementary Table S5. The top 50 cold-induced genes in *Arabidopsis thaliana* at 10°C treatment, determined by their LogFC values relative to the 22°C control condition.

No.	Gene ID	Protein	LogFC	Description
1	At3g63460	SEC31B	11.4	Protein transport protein SEC31 homolog B
2	At1g05200	GLR3.4	11.4	Glutamate receptor 3.4
3	At4g25450	ABCB28	11.2	ABC transporter B family member 28
4	At5g47430	AT5G47430	10.8	E3 ubiquitin ligase PQT3-like
5	At1g21380	TOL3	10.8	TOM1-like protein 3
6	At4g16990	RLM3	10.8	Disease resistance protein RLM3
7	At1g11720	SS3	10.7	Starch synthase 3
8	At3g17609	HYH	10.7	Transcription factor HY5-like
9	At1g80270	PPR596	10.6	Pentatricopeptide repeat-containing protein
10	At4g26530	FBA5	10.6	Fructose-bisphosphate aldolase 5
11	At3g57660	NRPA1	10.6	DNA-directed RNA polymerase I subunit 1
12	At3g50380	AT3G50380	10.2	Vacuolar protein sorting-associated protein 13b
13	At4g31210	AT4G31210	10.1	DNA topoisomerase 1
14	At5g62570	CBP60A	9.8	Calmodulin-binding protein 60 A
15	At2g02570	SPF30	9.7	Survival of motor neuron-related-splicing factor 30
16	At1g01060	LHY	9.1	Late elongated hypocotyl
17	At2g42540	COR15A	9.0	Protein COLD-REGULATED 15A
18	At3g25840	PRP4KA	8.9	Serine/threonine-protein kinase PRP4 homolog
19	At5g20830	SUS1	8.8	Sucrose synthase 1
20	At3g55610	P5CS2	8.5	Delta-1-pyrroline-5-carboxylate synthase B
21	At2g23420	NAPRT2	8.5	Nicotinate phosphoribosyltransferase 2
22	At2g46440	CNGC11	8.4	Cyclic nucleotide-gated ion channel 11
23	At3g54500	LNK2	8.4	Night light-inducible and clock-regulated gene 2
24	At5g17030	UGT78D3	8.3	UDP-glycosyltransferase 78D3
25	At4g00710	BSK3	8.3	Serine/threonine-protein kinase BSK3
26	At5g38410	RBCS3B	8.3	Ribulose bisphosphate carboxylase small chain 3B
27	At1g48540	AT1G48540	8.2	Outer arm dynein light chain 1, protein kinase binding
28	At2g36850	GSL8	8.1	Callose synthase 10
29	At1g03080	NET1D	7.7	Protein NETWORKED 1D
30	At3g51240	F3H	7.5	Flavanone 3-hydroxylase
31	At4g14690	ELIP2	7.4	Early light-induced protein 2
32	At3g63340	AT3G63340	6.7	Probable protein phosphatase 2C 51

33	At4g01985	AT4G01985	6.7	Uncharacterized protein
34	At5g37130	AT5G37130	6.7	Tetratricopeptide repeat protein 27 homolog
35	At5g27970	AT5G27970	6.5	Uncharacterized protein, ARM repeat superfamily protein
36	At4g04850	KEA3	5.9	K(+) efflux antiporter 3
37	At1g61800	GPT2	5.9	Glucose-6-phosphate/phosphate translocator 2
38	At5g37260	RVE2	5.8	MYB family transcription factor Circadian 1
39	At5g35210	PTM	5.8	PHD type transcription factor with transmembrane domains
40	At5g22760	DDP2	5.7	PHD finger family protein
41	At1g34260	FAB1D	5.6	Putative 1-phosphatidylinositol-3-phosphate 5-kinase
42	At1g30620	MUR4	5.6	UDP-arabinose 4-epimerase 1
43	At3g22840	ELIP1	5.5	Early light-induced protein 1
44	At4g36980	AT4G36980	5.4	CLK4-associating serine/arginine rich protein
45	At5g52310	LTI78	5.3	Low-temperature-induced 78 kDa protein
46	At1g60260	BGLU5	5.2	Beta glucosidase 5
47	At4g12735	AT4G12735	5.2	Hypothetical protein, unknown protein, peroxisomal protein
48	At2g04050	DTX3	5.1	MATE efflux family protein, Protein DETOXIFICATION 3
49	At4g30990	AT4G30990	5.0	ARM repeat superfamily protein
50	At3g54350	emb1967	5.0	Forkhead-associated (FHA) domain-containing protein

Supplementary Table S6. Genes in the 'RNA secondary structure unwinding' category of upregulated biological processes (BPs).

No.	Gene ID	Protein	0°C	4°C	10°C	Description
1	At3g01540	RH14	3.8	3.5	2.2	DEAD-box ATP-dependent RNA helicase 14
2	At1g54270	IF4A2	3.5	2.8	1.9	Eukaryotic initiation factor 4A-2
3	At5g14610	RH46	3.4	3.9	3.2	DEAD-box ATP-dependent RNA helicase 46
4	At2g21660	RBG7	3.3	2.8	0.4	Glycine-rich RNA-binding protein 7
5	At3g58570	RH52	3.1	2.7	2.7	DEAD-box ATP-dependent RNA helicase 52
6	At5g63120	RH30	3.1	3.2	2.5	DEAD-box ATP-dependent RNA helicase 30
7	At3g13920	IF4A1	2.9	2.1	2.2	Eukaryotic initiation factor 4A-1
8	At3g16840	RH13	2.8	2.7	2.2	DEAD-box ATP-dependent RNA helicase 13
9	At3g22310	RH9	2.6	3.0	2.7	DEAD-box ATP-dependent RNA helicase 9
10	At4g34910	RH16	2.5	2.3	1.9	DEAD-box ATP-dependent RNA helicase 16
11	At4g36020	CSP1	2.5	2.1	1.7	Cold shock protein 1
12	At2g42520	RH37	2.4	1.1	0.1	DEAD-box ATP-dependent RNA helicase 37
13	At1g63250	RH48	2.2	2.8	2.4	DEAD-box ATP-dependent RNA helicase 48
14	At4g16630	RH28	2.1	2.7	2.4	DEAD-box ATP-dependent RNA helicase 28
15	At5g54910	RH32	2.1	2.4	1.9	DEAD-box ATP-dependent RNA helicase 32
16	At3g18600	RH51	2.1	3.0	2.9	DEAD-box ATP-dependent RNA helicase 51
17	At3g09720	RH57	2.0	2.4	2.2	DEAD-box ATP-dependent RNA helicase 57
18	At1g77030	RH29	2.0	2.7	2.5	DEAD-box ATP-dependent RNA helicase 29
19	At1g51380	RH34	1.9	2.9	3.0	DEAD-box ATP-dependent RNA helicase 34
20	At3g58510	RH11	1.8	1.9	1.2	DEAD-box ATP-dependent RNA helicase 11
21	At5g08620	RH25	1.6	2	2.1	DEAD-box ATP-dependent RNA helicase 25
22	At2g40700	RH17	1.3	2.1	1.8	DEAD-box ATP-dependent RNA helicase 17

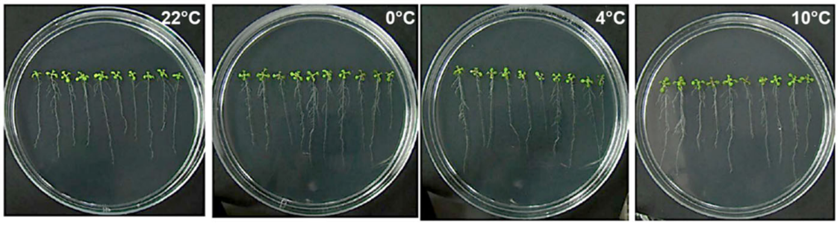

Supplementary Figure S1. *Arabidopsis thaliana* plantlets exposed to cold stress. Representative plates of 14-day-old plantlets after being exposed to low temperatures (0°C, 4°C, and 10°C), and a control temperature (22°C), for 24 h. Groups of ten plantlets were placed in each Petri dish for each biological replicate (*n*=3), and they were subsequently subjected to RNA extraction and RNA sequencing (RNA-seq).

A

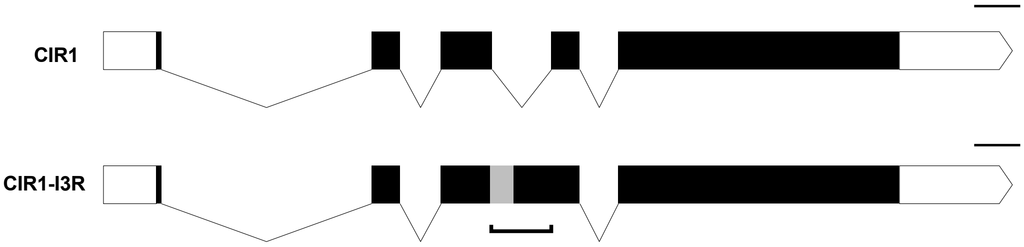

B

CIR1 MAMQERCESLCSDELISSSDAFYLKTRKPY**TITKQREKWTEAEHEKFVEALKLYGRAWRRIEEHVGTKTAVQIRSHAQKFFT KVA**RDFGVSSESIEIPPPRPKRKPMHPYPRKLVIPDAKEMVYAELTGSKLIQDEDNRSPTSVLSAHGSDGLGSIGSNSPNSSSA ELSSHTEESLSLEAETKQSLKLFGKTFVVGDYNSSMSCDDSEDGKKKLYSETQSLQCSSSTSENAETEVVVSEFKRSERSAF SQLKSSVTEMNNMRGFMPYKKRVKVEENIDNVKLSYPLW

CIR1-I3R MAMQERCESLCSDELISSSDAFYLKTRKPY**TITKQREKWTEAEHEKFVEALKLYGRAWRRIE**GVCAYLTLESLMHILI

C

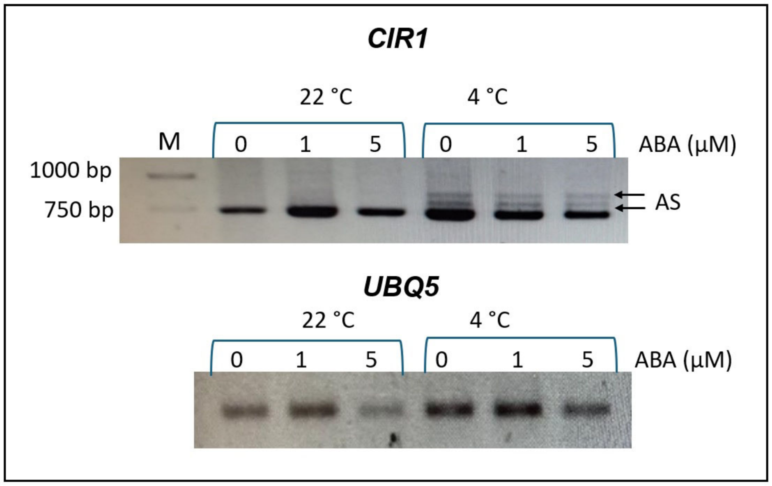

Supplementary Figure S2. Genomic organization of the *CIR* gene and its alternatively spliced version CIR1-I3R, along with their amplification by RT-PCR.

a) A schematic diagram of the genomic organization of the *CIR1* gene, illustrating the UTRs (white boxes), exons (black boxes), and introns (lines), with a scale bar indicating 100 nt. Another schematic depicts the intron-retained version *CIR1*-I3R, where the retention of intron 3 is highlighted by a bracket. The alternative splicing event leads to the introduction of a premature stop codon within the retained intron 3. The gray box represents the region that becomes part of the new coding sequence until the stop codon in intron 3. b) Amino acid sequence of the CIR1 protein (287 amino acids), with the MYB domain highlighted in bold. The predicted amino acid sequence of the intron-retained version (*CIR1-I3R*) results in a shorter protein of 78 amino acids, with 62 amino acids corresponding to exons 1 through 3, and an additional 16-amino acid peptide encoded by the intron. c) Expression analysis of *CIR1* and its alternative splicing versions. RT-PCR was performed on 14-day-old *Arabidopsis* plants under the following treatments: 4ºC, 4ºC + 1µM ABA, 4ºC + 5µM ABA, 22ºC, 22ºC + 1µM ABA, and 22ºC + 5µM ABA. PCR products were separated on agarose gels: 1.5% for the *CIR1* gene and 1%for the UBQ5 gene. AS: alternative splicing (arrows).

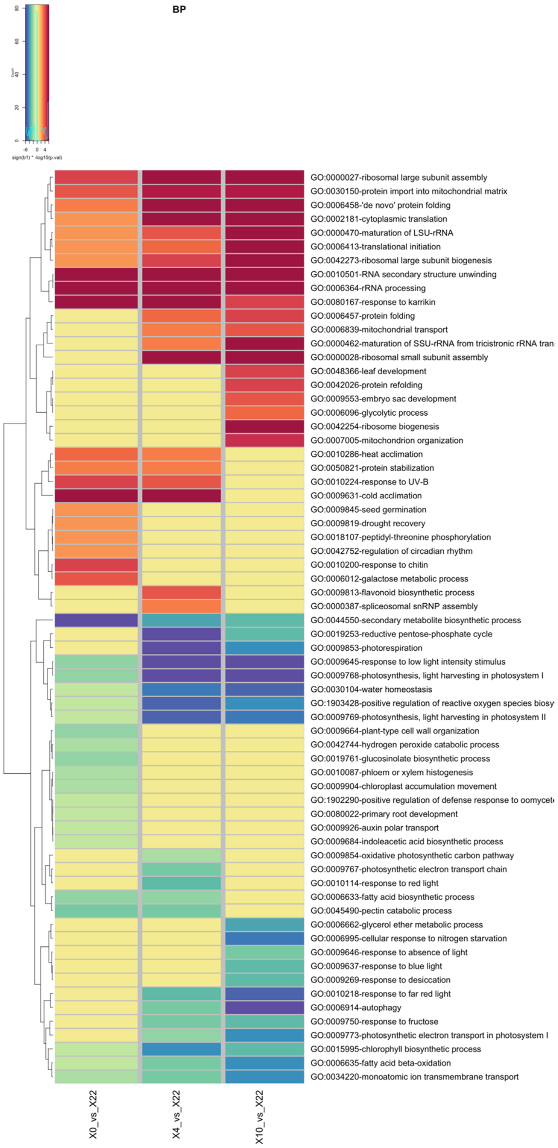

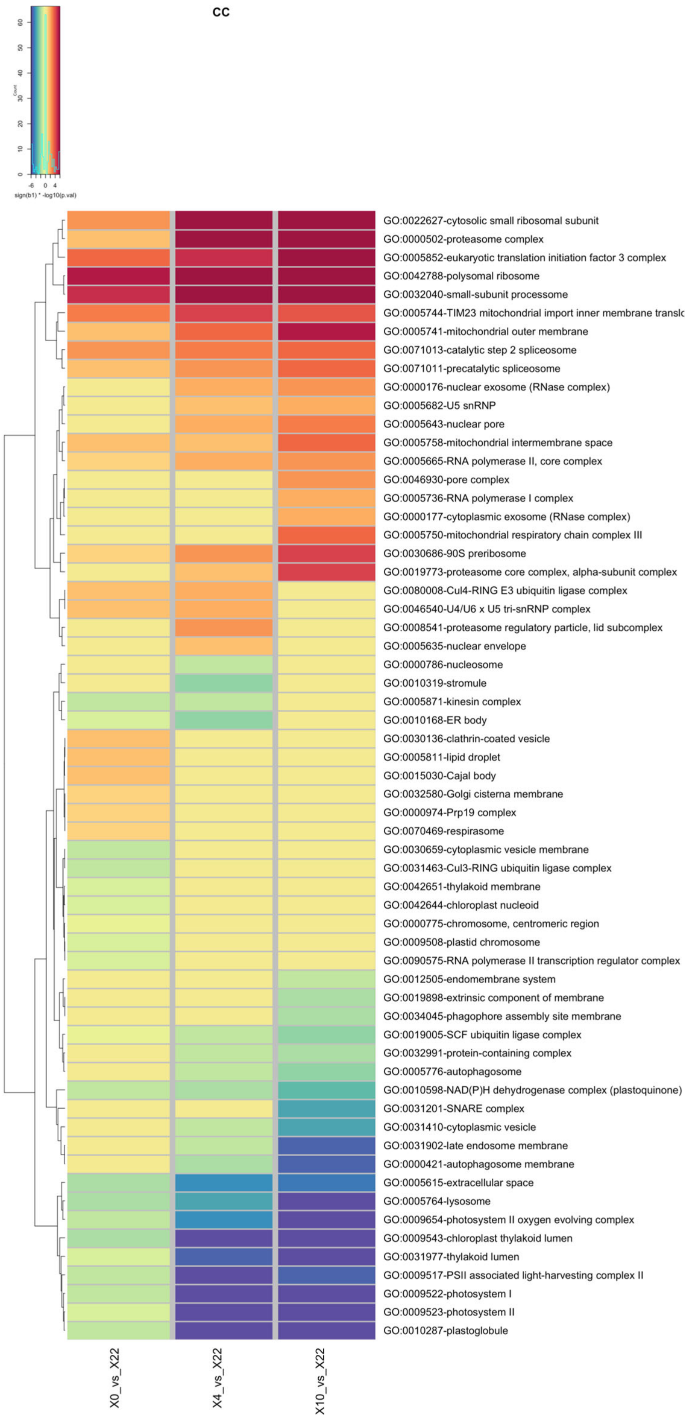
CC
GO:0022627-cytosolic small ribosomal subunit
GO:0000502-proteasome complex
GO:0005852-eukaryotic translation initiation factor 3 complex
GO:0042788-polysomal ribosome
GO:0032040-small-subunit processome
GO:0005744-TIM23 mitochondrial import inner membrane translc
GO:0005741-mitochondrial outer membrane
GO:0071013-catalytic step 2 spliceosome
GO:0071011-precatalytic spliceosome
GO:0000176-nuclear exosome (RNase complex)
GO:0005682-U5 snRNP
GO:0005643-nuclear pore
GO:0005758-mitochondrial intermembrane space
GO:0005665-RNA polymerase II, core complex
GO:0046930-pore complex
GO:0005736-RNA polymerase I complex
GO:0000177-cytoplasmic exosome (RNase complex)
GO:0005750-mitochondrial respiratory chain complex III
GO:0030686-90S preribosome
GO:0019773-proteasome core complex, alpha-subunit complex
GO:0080008-Cul4-RING E3 ubiquitin ligase complex
GO:0046540-U4/U6 x U5 tri-snRNP complex
GO:0008541-proteasome regulatory particle, lid subcomplex
GO:0005635-nuclear envelope
GO:0000786-nucleosome
GO:0010319-stromule
GO:0005871-kinesin complex
GO:0010168-ER body
GO:0030136-clathrin-coated vesicle
GO:0005811-lipid droplet
GO:0015030-Cajal body
GO:0032580-Golgi cisterna membrane
GO:0000974-Prp19 complex
GO:0070469-respirasome
GO:0030659-cytoplasmic vesicle membrane
GO:0031463-Cul3-RING ubiquitin ligase complex
GO:0042651-thylakoid membrane
GO:0042644-chloroplast nucleoid
GO:0000775-chromosome, centromeric region
GO:0009508-plastid chromosome
GO:0090575-RNA polymerase II transcription regulator complex
GO:0012505-endomembrane system
GO:0019898-extrinsic component of membrane
GO:0034045-phagophore assembly site membrane
GO:0019005-SCF ubiquitin ligase complex
GO:0032991-protein-containing complex
GO:0005776-autophagosome
GO:0010598-NAD(P)H dehydrogenase complex (plastoquinone)
GO:0031201-SNARE complex
GO:0031410-cytoplasmic vesicle
GO:0031902-late endosome membrane
GO:0000421-autophagosome membrane
GO:0005615-extracellular space
GO:0005764-lysosome
GO:0009654-photosystem II oxygen evolving complex
GO:0009543-chloroplast thylakoid lumen
GO:0031977-thylakoid lumen
GO:0009517-PSII associated light-harvesting complex II
GO:0009522-photosystem I
GO:0009523-photosystem II
GO:0010287-plastoglobule
X0_vs_X22
X4_vs_X22
X10_vs_X22

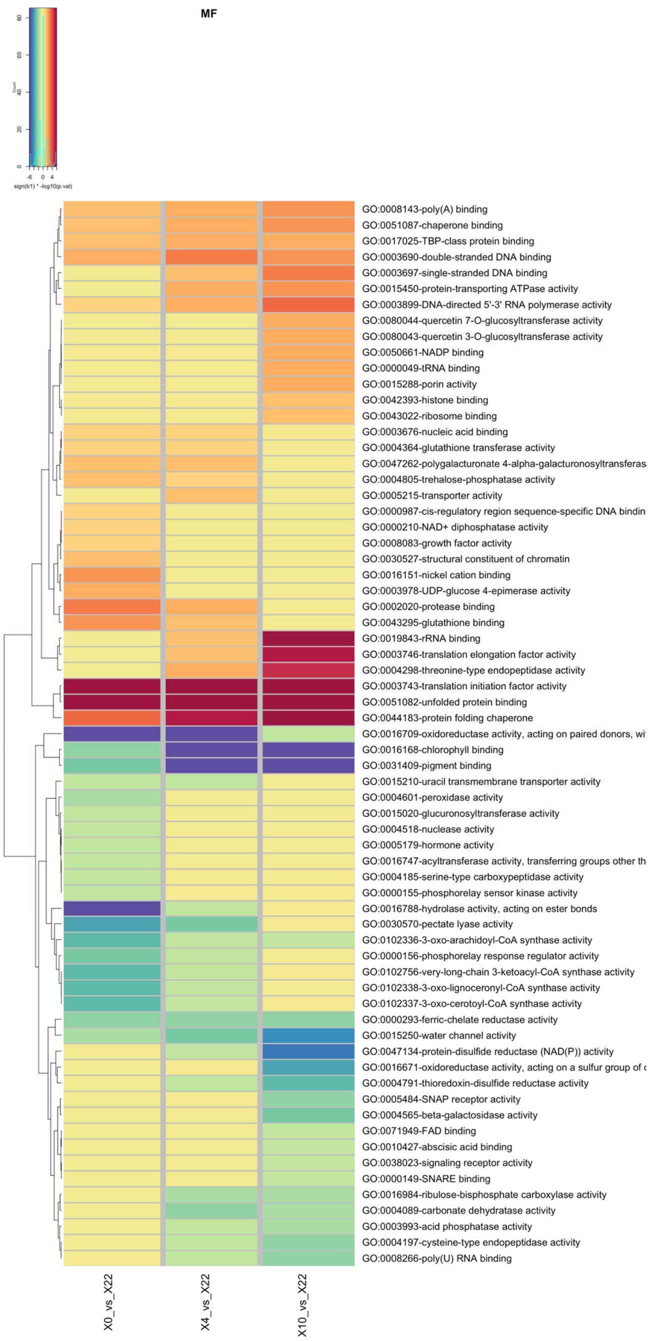

Supplementary Figure S3. Gene ontology enrichment analysis of genes regulated by cold stress. The heatmap displays the main Biological Processes (BPs), Cellular Components (CC), and Molecular Functions (MF) that were up-regulated in varying shades of red and orange, down-regulated in shades of blue and green, and unchanged in yellow. The RNA-seq analysis was conducted on 14-day-old plantlets exposed to low temperatures (0°C, 4°C, and 10°C) compared to a control temperature of 22°C. The *p*-values and enrichment signs are represented by the color scale in the heatmap (color = sign(enrichment) * -log10(*p*-value)).

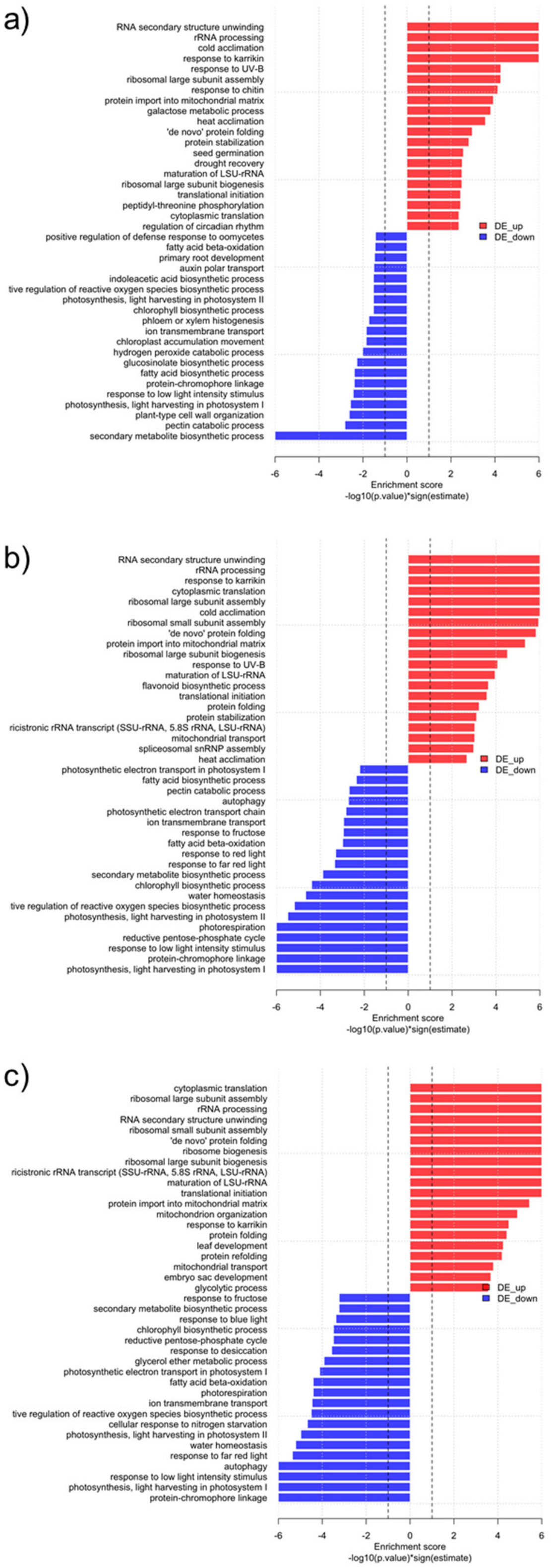

Supplementary Figure S4. Enriched biological processes (BP) are indicated in red (upregulated) and blue (downregulated) at temperatures a) 0°C, b) 4°C, and c) 10°C.
